# Cavernome du quatrième ventricule: à propos d'un cas et revue de la literature

**DOI:** 10.11604/pamj.2014.18.331.5193

**Published:** 2014-08-25

**Authors:** Fahd Derkaoui Hassani, Rachid Gana, Najia El Abbadi, Nizare El Fatemi, Moulay Rachid Maaqili

**Affiliations:** 1 Service de Neurochirurgie, Université Mohammed V Souissi, CHU Ibn Sina, Rabat, Maroc

**Keywords:** Cavernome, ventricule cérébraux, encéphale, Cavernoma, berebral ventricule, brain

## Abstract

Le siège intraventriculaire est une localisation rare des cavernomes de l'encéphale. Le quatrième ventricule est le moins concerné de toutes les localisations. Nous rapportons le cas d'une patiente âgée de 52 ans qui présente depuis 12 mois un syndrome d'hypertension intracrânienne et trouble de l’équilibre. Elle s'est présentée aux urgences avec un GCS à 14, nuque subraide, un syndrome cérébelleux statokinétique avec une acuité visuelle basse et un oedeme papillaire bilatéral. Une TDM cérébrale réalisée aux urgences a objectivé un hématome du 4ème ventricule avec hydrocéphalie active triventriculaire. Une dérivation ventriculaire interne a été réalisée en urgence avec une bonne évolution clinique post opératoire. Le bilan a été complété par une IRM cérébrale objectivant un processus du quatrième ventricule évoquant un cavernome. Un abord direct a été réalisé permettant une exérèse totale du cavernome siégeant au sein du quatrième ventricule. L'anatomo-pathologie a confirmée le diagnostic. A notre connaissance, il s'agit du 13^ème^ cas rapporté dans la littérature. Les cavernomes intraventriculaires représentent 2,5-10% de tous les cavernomes dont 9% est au niveau 4ème ventricule. Les patients sont souvent admis aux urgences suite au saignement de cette malformation angiomateuse. Le diagnostic est rendu accessible par les différentes séquences de l'IRM. Le traitement est souvent chirurgical vu le risque de resaignement. Le pronostic dépend de l’état initial du patient et de l'infiltration du plancher du quatrième ventricule.

## Introduction

Le siège intraventriculaire est une localisation rare des cavernomes de l'encéphale. Le quatrième ventricule est le moins concerné de toutes les cavités Il s'agit du 13^ème^ cas dans la littérature après une recherche exhaustive depuis sa première description en 1905.

## Patient et observation

**Figure 1:**
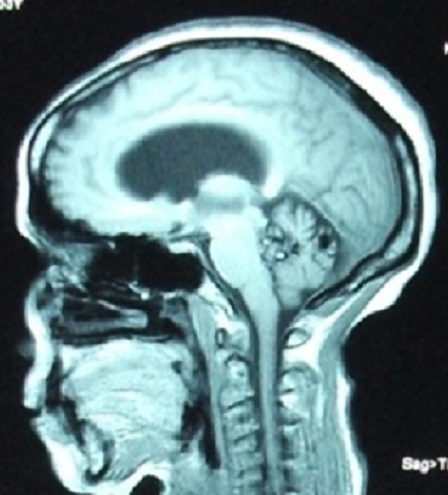
Coupe sagittale d'une IRM encéphalique en séquence T1 sans injection de produit de contraste objectivant un processus hétérogène siégant au niveau du quatrième ventricule avec un contact intime avec son plancher

**Figure 2:**
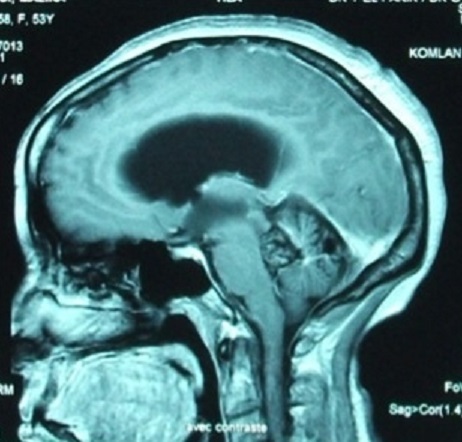
IRM encéphalique en coupe sagittale en séquence T1 avec injection de produit de contraste ne révele pas de prise de contraste au sein de la lésion en intraventriculaire ni au niveau du tronc cerebral

**Figure 3:**
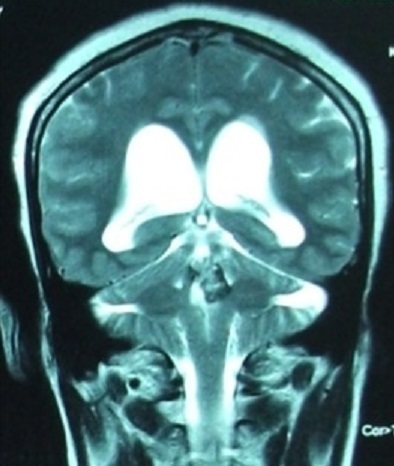
Aspect hétérogène d'un processus du quatrième ventricule en séquence T2

**Figure 4:**
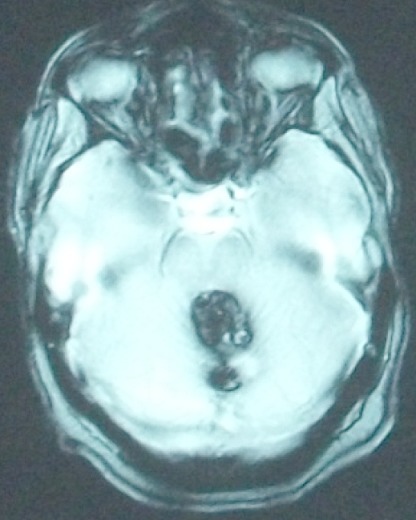
Séquence d’écho de gradient en coupe axiale met en évidence l'aspect en « poivre et sel » typique d'un cavernome du quatrième ventricule et un saignement au niveau du vermis

Nous rapportons le cas d'une patiente âgée de 52 ans qui présente depuis 12 mois un syndrome d'hypertension intracrânienne et trouble de l’équilibre. Elle s'est présentée aux urgences avec un GCS à 14, nuque subraide, un syndrome cérébelleux statokinétique et un oedème papillaire bilatéral. Une TDM cérébrale réalisée aux urgences a objectivé un hématome du quatrième ventricule avec hydrocéphalie active triventriculaire. Une dérivation ventriculo-péritonéale a été réalisée en urgence avec une bonne évolution clinique post opératoire. Le bilan a été complété par une IRM cérébrale (Figure 1, Figure 2, Figure 3) objectivant une lésion du quatrième ventricule hétérogène en séquence T1 et T2 avec contact intime avec le plancher du 4ème ventricule correspondant à son infiltration. La séquence en écho de gradient permet d'apprécier l'aspect « poivre et sel » typique d'un cavernome du quatrième ventricule (Figure 4). Un abord sous occipital sous tonsillaire a été réalisé permettant d'objectiver un processus siégeant au sein du 4ème ventricule, d'aspect noir rougeâtre qui infiltre le plancher du 4ème ventricule. On procéde par une dissection du cavernome qu'on suit jusqu'au niveau du plancher du quatrième ventricule. Une dissection délicate du cavernome du plancher du quatrième ventriucle et sa dévascularisation permettent de réaliser une exérèse totale en bloc du cavernome. L'anatomo-pathologie a confirmée le diagnostic d'un cavernome. L’évolution a été marquée par une amélioration initiale en post opératoire; puis la patiente a présenté une pneumopathie nosocomiale avec réadmission à la réanimation. La patiente est décédée 4 semaines en post opératoire dans un tableau de choc septique.

## Discussion

Les cavernomes intraventriculaires représentent 2,5 - 10% de toutes les localisations des cavernomes [1, 2]. Le cavernome du quatrième ventricule est une entité exceptionnelle par sa localisation. Sur 89 cas de cavernomes intraventriculaires, rapportés sur un siècle, cette localisation représente 9%; ce qui représente la localisation la plus rare des cavernomes. [2] La manifestation clinique la plus rencontrée est un syndrome d'hypertension intracrânienne parlant d'une hydrocéphalie triventriculaire d'origine obstructive par le processus ou compliquant un épisode hémorragique. [2] Notre patiente était admise dans un tableau de préengagment compliquant un épisode hémorragique du quatrième ventricule. L'IRM cérébrale permet souvent le diagnostic [2]. Sur les séquences pondérées T1 et T2, le centre parait avec un signal hétérogène, alors que la périphérie parait en hyposignal ce qui suggère un cercle d'hémosidérine témoignant d'un saignement répété de la lésion. La séquence pondérée T1 avec injection de gadolinium permet d'avoir une prise de contraste de ce processus pouvant varier d'un rehaussement important jusqu’à l'absence de prise de contraste. La séquence d'IRM pondérée en Echo de gradient est la séquence maitresse dans le diagnostic des cavernomes. Aussi on apprécie sur cette IRM la présence d'effet de masse, d'une hydrocéphalie sus-jacente, et parfois de peu d'oedème perilésionnel [2].

L'histoire naturelle des cavernomes est marquée par une tendance au resaignement. La littérature relève seulement que 14% des cas publies de cavernomes intraventriculaires se sont présentés pour une hémorragie intraventriculaire [1]. Généralement; le resaignement des cavernomes intraventriculaires ne constituent pas de façon significative un risque pour un déficit neurologique permanent. Sur les 89 cas publiés des cavernomes intraventriculaires [1], seulement un patient est décédé avant toute chirurgie d'exérèse suite à un resaignement du cavernome dans la corne temporale du ventricule latéral avec une inondation ventriculaire massive et un hématome intracérébral. La chirurgie est toujours indiquée chaque fois que la lésion est accessible et qu'il faille rétablir la circulation du LCR.

A notre connaissance, depuis la première description d'un cavernome du quatrième ventricule en 1905, 12 cas (Tableau 1) ont été rapportés [3–10]. La médiane d’âge est de 43,5 ans, avec une prédominance féminine à 2:1. 5 patients se sont présentés avec une hémorragie ventriculaire. Tous les patients ont été opérés. 9 ont bénéficié d´une exérèse totale avec une amélioration post opératoire à long terme. 3 patients ont eu une exérèse partielle dont 2 sont décédés en post opératoire et le troisième patient a eu une récidive des symptômes. Aucun décès n´a été note chez les patients avec une hémorragie intra ventriculaire, alors que l´exérèse partielle représente un éventuel un facteur de mauvais pronostic. La rareté de cette localisation rend délicate la vérification de cette hypothèse.

**Tableau 1:** Liste des 12 cas rapportés sur la littérature de cavernomes du quatrième ventricule de 1905 à 2012

	Cas rapportés	Age	Sexe	Tableauau clinique	Exérèse chirurgicale	Evolution
**1**	Finkelburg, 1905	2	M	DEFICIT	PARTIELLE	DECES
**2**	Dandy, 1928	31	M	DEFICIT	TOTALE	AMELIORATION
**3**	Giombini & Morello, 1978	27	M	DEFICIT	PARIELLE	DECES
**4**	Terao et al. 1979	29	F	HIV	TOTALE	AMELIORATION
**5**	Kendall et al. 1983	60	F	DEFICIT	PARTIELLE	RECIDIVE DES SYMPTOMES
**6**	Yamasaki et al. 1986	47	F	DEFICIT	TOTALE	AMELIORATION
**7**	Itoh & Usui, 1991	44	F	HIV ET DEFICIT	TOTALE	AMELIORATION
**8**	Kivelev, 2010	43	F	DEFICIT	TOTALE	AMELIORATION
**9**	Kivelev, 2010	58	F	HIV ET DEFICIT	TOTALE	AMELIORATION
**10**	Kivelev, 2010	15	M	HIV ET DEFICIT	TOTALE	AMELIORATION
**11**	Kivelev, 2010	49	F	DEFICIT	TOTALE	AMELIORATION
**12**	Kivelev, 2010	49	F	HIV ET DEFICIT	TOTALE	AMELIORATION
**13**	Notre cas	52	F	HIV ET DEFICIT	TOTALE	DECES

Sur une autre série de 12 cavernomes intraventriculaires rapportée par Kivelev et al [6], 5 ont été pris en charge pour une localisation au sein du quatrième ventricule dont deux ont présentés un resaignement avant la chirurgie expliqué probablement par un délai prolongé avant la chirurgie secondaire à une première hospitalisation au niveau de la structure référant le patient. Sur l'imagerie, le cavernome se situe sur la partie médiane du plancher du quatrième ventricule. L'ensemble des 5 patients ont bénéficié d'une craniotomie sous occipitale en position assise avec un abord sous tonsillaire. Une dissection délicate de la lésion du plancher du quatrième ventricule avec exérèse du bourgeon infiltrant le plancher est préconisée. En comparaison avec les 7 cas de cavernomes du ventricule latéral, la localisation au sein du quatrième ventricule est accompagnée d'un pronostic moins bon avec des séquelles neurologiques plus importantes et plus fréquentes très probablement à cause de la localisation à la fois au niveau du quatrième ventricule mais surtout à cause de l'infiltration du tronc cérébral.

## Conclusion

Le carvernome du quatrième ventricule est une entité rare. Nous rapportons un cas devant la rareté et la particularité du traitement et des résultats. L'hémorragie intraventriculaire n'est pas synonyme de mauvais pronostic. Ce dernier dépend de la localisation et de l'infiltration du plancher du quatrième ventricule.
